# Analyzing and simulating heat transfer and designing a shell and tube heat exchanger for the pasteurization process of tomato paste: A CFD study

**DOI:** 10.1016/j.heliyon.2023.e21593

**Published:** 2023-10-31

**Authors:** Sh. Asadbeigi, E. Ahmadi, M. Goodarzi, A. Sagharichian

**Affiliations:** a Department of Biosystems Engineering, Bu-Ali Sina University, Hamadan, Iran; b Department of Mechanical Engineering, Bu-Ali Sina University, Hamadan, Iran; c Department of Biosystems Engineering, Tarbiat Modares University, Tehran, Iran

**Keywords:** Computational fluid dynamics, Shell and tube heat exchanger, Pasteurization, Tomato paste, Herschel-Bulkley model

## Abstract

Pasteurization is a vital process to destroy harmful enzymes. This process is very critical to obtain quality tomato paste. In this study, Computational Fluid Dynamics (CFD) has been used to design a shell and tube heat exchanger on an industrial scale and to simulate heat transfer in order to visualize this process and present it to the industry. In this research, a three-dimensional CFD model was simulated using ANSYS FLUENT commercial software. Also, using the Herschel-Bulkley model, the behavior of viscosity in the pasteurization process of tomato paste has been explained. In this stage of the production line, the tomato paste enters a shell and tube heat exchanger at 65 °C and reaches 80 °C at the outlet. Compared with the experimental data, the output temperature of tomato paste predicted by CFD simulation reached 79 °C. In addition, thermophysical properties of tomato paste were measured, and these exact values were used for simulation. Also, the evaluation of this heat exchanger with three hot water inlet mass flow rates has been done in order to provide the results to the factory to avoid spending more energy. And the simulation results showed that the output temperature of tomato paste at three different mass flow rates did not change less than the mass flow rates measured in the factory, and also the output visualizations from this research can be suitable for presenting to the industry and benefiting from them.

## Highlights

• Computational fluid dynamics (CFD) is the science of predicting fluid flow.
• Shell and tube heat exchanger is the most common type of exchanger in industries.
• Tomato is one of the agricultural products that is widely used (sauce and paste).
• No study has investigated tomato paste production line using CFD.

## Graphical abstract

**Figure:**
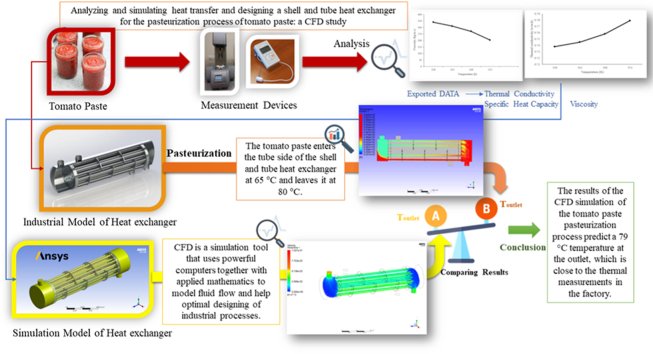

## Nomenclature

P: Pressure, Pa
T: Temperature, °C
U: Fluid velocity, m s^−1^
ρ: Density, Kg m^−3^
$\tau_{0}$: Yield stress, N m ^−2^
n: Flow behaviour index
ν: fluid kinematic viscosity, M^2^ s^−1^
Pr: Prandtl number
Γ: generation of turbulence kinetic energy k
d: Diameter of the tubes
μ: dynamic viscosity., Pa s
k: Consistency factor

## 1 Introduction

Tomato (Solanum lycopersicum) represents a good source of essential nutrients and bioactive compounds such as vitamins, carotenoids, and phenolic compounds, which contribute significantly to human health. Tomato is full of carotenoids that are beneficial to health. The antioxidant and chemotherapy-preventing properties are the most important effects of lycopene, beta-carotene, and other carotenoids existing in tomatoes [1].

Tomato is used as a fresh vegetable or processed product such as tomato paste, concentrate, catchup, and sauce [2,3]. Tomato paste, which is used as an ingredient in many products such as soups, sauces, and Ketchup [4]. As a result, ensuring the preservation of these compounds after processing is so important to maintain the tomato's health effects [5]. Industrial tomato processing consists of thermal stages such as pasteurizing, heating, and drying. These operations stabilize the product by inactivating enzymes and microorganisms [6]. These thermal operations can dissociate bioactive compounds and reduce the ascorbic acid and lycopene concentrations in the tomato processed products [7]. Therefore, designing and optimizing these thermal operations in food production lines, especially the tomato, can lead to a high-quality product from the perspective of color, taste, and nutrients. In the food industry, heat treatment of fluid foods in a shell and tube heat exchanger is a common application. Pasteurization is one of such applications. It is a food preservation technique that uses a high-temperature treatment. Depending on the necessity, fluid foods are heated to a temperature. For the thermal treatment of fluid foods such as milk, hazy orange juice, and apples, heat exchangers are utilized [8]. The shell and tube heat exchanger is the most common heat exchanger in chemical industries, petroleum refining processes, power plants, and the food industry. This exchanger is used to transfer heat between two fluids [9]. Their widespread applications are the result of their simple design and low manufacturing cost [10]. The main purpose of this device is to make foods and beverages safe to consume by eliminating the microbials and increasing their shelf life. In the food industry, this device is mostly preferred because of the easy cleaning and sanitizing properties of the surface. The exchanger is designed based on the viscosity and size of food materials that are being used and the industrial needs [11]. These heat exchangers consist of many tubes inside a coaxial shell. Heat transfer occurs when a fluid flows in the tubes while another fluid flows inside the shell side. Baffles are used to distribute fluid flow inside the shell and also to increase heat transfer. Therefore, the shape and structure of baffles are critical for heat exchanger operation. Computational fluid dynamics (CFD) has widespread applications in food processing for the design and optimization of equipment such as ovens, driers, chillers, and heat exchangers. CFD is a useful tool for predicting flow, velocity, and temperature distribution patterns.

CFD is a simulation tool that uses powerful computers together with applied mathematics to model fluid flow and help optimal designing of industrial processes. This method includes solving mass, momentum, and energy conservation equations [12]. CFD has enormous potential and many opportunities to improve the quality and safety of food products, as well as to reduce the costs of production and the use of machines and production equipment. Despite some disadvantages, such as the need to have a large reserve of computing power, the development of digital and IT technologies will make this problem insignificant in the near future. The advantage of using CFD in this research is to use the property of process visualization to present to the industry while taking advantage of reducing maintenance costs [13].

Numerous benefits are reported in CFD applications in the food industry. Plate heat exchangers have widespread applications in dough pasteurization, heat transfer, and hydrodynamic simulation of these heat exchangers for milk. These studies clearly show the parts of the heat exchanger that are in higher temperature than the other parts [[14], [15], [16]]. In addition, several studies reported the design and analysis of different baffles with various mass flow rates for shell and tube heat exchangers [[17], [18], [19]]. In these investigations, the solid-liquid food mixture and canned food in the sterilization process are numerically simulated [[20], [21], [22]]. [23] analyzed the pasteurization process of milk using CFD. They found that CFD can be used as a tool to determine the temperature distribution pattern in canned milk during the thermal process [24]. simulated the pasteurization process of canned fruit salad in a glass. In this investigation, samples of fruit salad consisting of five different fruits with various shapes, sizes, and thermal properties are included in Fluent. The experimental results showed that the lowest thermal point is located at 19–20 % of the height of the can. The CFD simulation results showed only a slightly different lowest thermal point for all the fruits compared to the experimental results [25]. used CFD to analyze air flow and heat transfer in apple shipping boxes inside the container. In this study, a rectangular container with square boxes inside, containing 12 apples, is simulated. Each container contained 8 apple boxes. In this investigation, the porous media technique was used for CFD simulation of the cooling process in a container. Using the simulation results, regions with low airflow between the boxes and high temperature are identified [26]. Investigated the design of a shell and tube heat exchanger using alumina-water nanofluids to improve heat transfer in food products, as well as changes in temperature-time profiles and energy consumption rates [27]. Have researched the effects of nanofluid in the thermal process of watermelon in a shell and tube heat exchanger. They found that substituting 4 % nanofluid instead of water can maintain a higher percentage of vitamin C and lycopene in this thermal process.

The industrial pasteurization process of tomato paste is performed by the shell and tube heat exchanger. This study aims to simulate heat transfer in the pasteurization process of tomato paste and to present a model describing the rheological behavior of tomato paste. The current research developed simulation CFD about the non-Newtonian fluid of tomato paste in the industrial model of shell and tube heat exchanger, while previous researches focused on laboratory models.

This process is done by the shell and tube heat exchanger in the tomato paste production line. The paste after concentration enters the pasteurization device. Direct heat in this process will burn the product and will increase waste which as a result, the quality and appearance of the tomato paste will deteriorate. To eliminate this problem, a shell and tube heat exchanger is used in which the paste flows in the tubes and hot water flows around them. Until now, no study has investigated this stage of tomato paste production line in the shell and tube heat exchanger using CFD. Therefore, it is a necessity to simulate this process, so accurate information will be provided to production line engineers and factories. The results of this research can be presented to the food industry to produce safe food. Because CFD can simulate other similar processes.

### 1.1 Model preparation and simulation

**Fig. 1:**
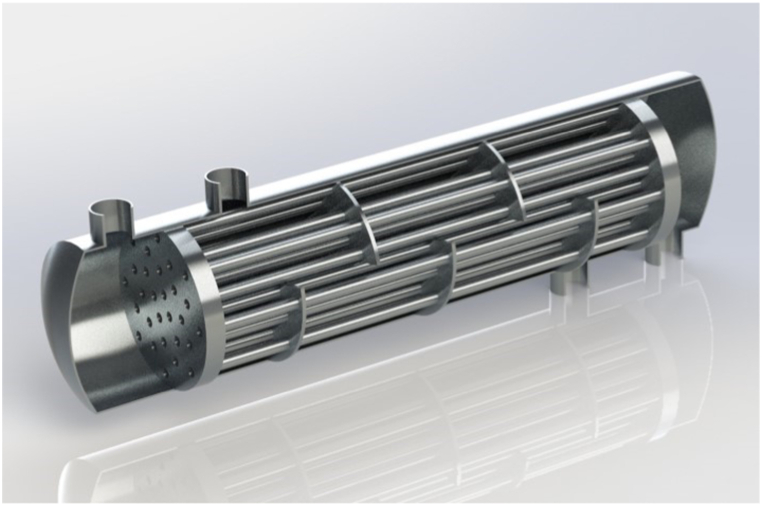
Modeling of shell and tube heat exchanger.

The geometry of the shell and tube heat exchanger is created in SOLIDWORKS version 2021. The design parameters for this heat exchanger are presented in Table 1. According to industrial models, the heat exchanger is 3.5 m in length and 273.5 kg in weight. Then, this geometry is entered into FLUENT 2020R1 for simulation. Fig. 1 demonstrates the overall view of the heat exchanger including baffles, tubes, and the shell after designing in the SOLIDWORKS software.

**Table 1:** Geometric parameters of shell and tube heat exchanger.

Parameter	Value	Unit
Shell diameter	82	Cm
Inner diameter of tubes	50	Mm
Outer diameter of tubes	60	Mm
Number of tubes	28	Pcs
Number of baffles	5	Pcs
Baffle cut	22	%

### 1.2 Boundary conditions

**Fig. 2:**
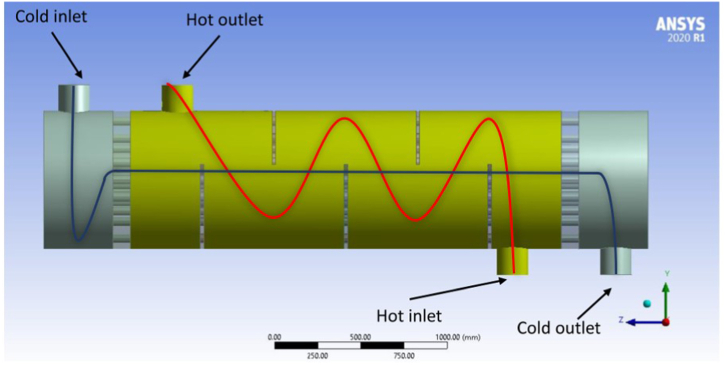
Isometrical view of the computational domains for shell and tube heat exchanger and flow paths in two domains.

The boundary conditions are defined as two fluid domains as the hot and cold domains. The tomato paste enters the tube side at 65 °C, and the hot water enters the shell side at 88 °C. Fig. 2 demonstrates the inlets and outlets of the heat exchanger. The tomato paste inlet is considered a cold inlet with a 1.5 kg/s mass flow rate, and the hot water inlet is considered a hot inlet with a 1 kg/s mass flow rate. These values are from industry reports on the mass velocities of both inputs to achieve the desired output temperature of tomato paste.

The heat exchanger outlets, i.e., the paste and water, are considered hot and cold outlets, respectively. The pressure outlet boundary condition is applied to both outlets, which is defined as zero outlet pressure. The no-slip and nonpermeable boundary condition is applied to the other solid parts of the heat exchanger including the baffles and shell. Zero heat flux is considered for the shell wall, inlets, and outlet nozzle. The fluid-solid interface is applied between the fluid and tube domains for heat transfer as two coupled interfaces. The governing equations in ANSYS FLUENT are used to calculate heat transfer between these two fluids. The Realizable k-ε turbulence model is used, and the governing equations are solved using the SIMPLE algorithm. A second-order discretization scheme is used for momentum, energy, and turbulence equations. The software default factors of 0.3, 0.7, 0.8, and 0.8 are applied to momentum, pressure, turbulent kinetic energy, and turbulent kinetic energy dissipation. This problem is solved in ANSYS 2020R1 in 3D with double precision, and the pressure-based method is used for solver settings. An ASUS core i7 with 40 GB RAM is used to solve this problem. The problem is solved in a steady state. Steady-State solver was used to simulate this study due to the residuals convergence of the equations used for solving.

Given the fluid completely fills the heat exchanger and the effects of body forces are negligible in this problem, the gravity acceleration is not activated in this problem. The energy setting is activated as heat transfer involves. The turbulent intensity and hydraulic diameter are assumed 5 %. The flow of tomato paste inside the tubes was considered laminar due to its high viscosity.

### 1.3 Meshing

**Fig. 3:**
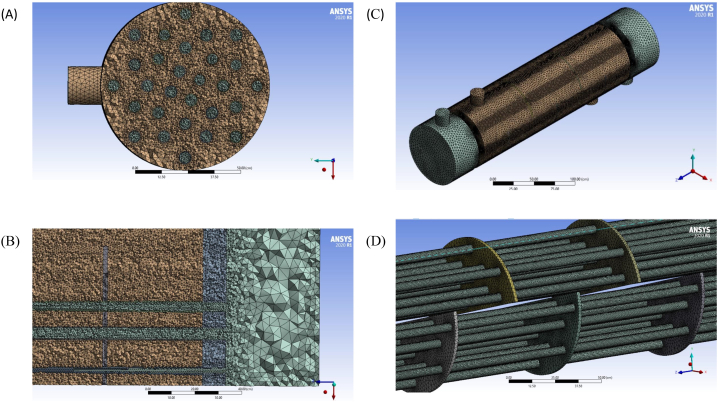
a) A sectional view of tubes, b) A sectional view of the meshing, c) Outside wall d) Meshing on baffles and tubes.

Unstructured meshes are widely used to delineate the complex geometry of in CFD simulation. Therefore, in this study, a high-quality, quadrilateral unstructured mesh with 8886046 elements of 3 cm size is used to discretize the domain. Fig. 3(a)-(d) illustrate different views of the mesh in various parts of the heat exchanger. Fig. 3(b) demonstrates node conformity at the interface between different parts of the model in ANSYS Meshing. The mesh demonstrates a good correlation between the size of the heat exchanger, the high quality of the mesh, and the number of elements. After introducing the model to the Fluent, the report quality option describes mesh quality. The minimum orthogonal quality measure must exceed 0.15. The current model conforms to this rule with a value of 1.52089e-01.

### 1.4 Governing equations

This model was based on the numerical solution of continuity in equation (1), momentum in equation (2), and energy in equation (3). Version 15 [28] is used to determine and write the governing equations for this problem.

#### 1.4.1 Continuity equation

(1) $$\frac{\partial u_{i}}{\partial x_{i}}=0$$

#### 1.4.2 Momentum equation

(2) $$\frac{\partial u_{i}u_{j}}{\partial x_{i}}=-\frac{1}{\rho }\frac{\partial p}{\partial x_{i}}+\frac{\partial }{\partial x_{i}}\left(\left(\nu +\nu_{turb}\right)\left(\frac{\partial u_{i}}{\partial x_{j}}+\frac{\partial u_{j}}{\partial x_{i}}\right)\right)$$

#### 1.4.3 Energy equation

(3) $$\frac{\partial u_{i}T}{\partial x_{i}}=\rho \frac{\partial }{\partial x_{i}}\left(\left(\frac{\nu }{\mathrm{Pr}}+\frac{\nu_{t}}{{\mathrm{Pr}}_{turb}}\right)\frac{\partial T}{\partial x_{i}}\right)$$

where P, T, and U represent the fluid velocity, temperature, and pressure, in equation (3) respectively. ρ is the fluid density, ν and Pr the fluid kinematic viscosity, and Prandtl number, subscript turb refers to turbulent. The Realizable k-ε model in equation (4) is used in this study since it exhibits a better performance with swirling flows and boundary layer flow under the adverse pressure gradient.

#### 1.4.4 Turbulent kinetic energy k equation

(4) $$\frac{\partial u_{i}k}{\partial x_{i}}=\frac{\partial }{\partial x_{i}}\left(\left(\nu +\frac{\nu_{t}}{\sigma_{k}}\right)\frac{\partial k}{\partial x_{i}}\right)+\Gamma -\varepsilon$$

#### 1.4.5 Turbulent energy dissipation

(5) $$\frac{\partial u_{i}\varepsilon }{\partial x_{i}}=\frac{\partial }{\partial x_{i}}\left(\left(\nu +\frac{\nu_{t}}{\sigma_{\varepsilon }}\right)\frac{\partial \varepsilon }{\partial x_{i}}\right)+c_{1}\Gamma \varepsilon -c_{2}\frac{\varepsilon^{2}}{k+\sqrt{\nu \varepsilon }}$$

where Γ in equation (5) represents the generation of turbulence kinetic energy k due to the mean velocity and given by equation (6):

(6) $$\Gamma =-\bar{u_{i}u_{j}}\frac{\partial u_{i}}{\partial x_{i}}=\nu_{turb}\left(\frac{\partial u_{i}}{\partial x_{j}}+\frac{\partial u_{j}}{\partial x_{i}}\right)\frac{\partial u_{i}}{\partial x_{i}}$$

The turbulent kinematic velocity in equation (7) is:

(7) $$\nu_{turb}=c_{\mu }\frac{k^{2}}{\varepsilon }$$

And according to the Reynolds equation in equation (8):

(8) $$Re=\frac{\rho ud}{\mu }$$

Where ρ, is the density, u is the fluid velocity, d is the diameter of the tubes and μ is the dynamic viscosity.

### 1.5 Physical properties of tomato paste

#### 1.5.1 Measuring viscosity

**Fig. 4:**
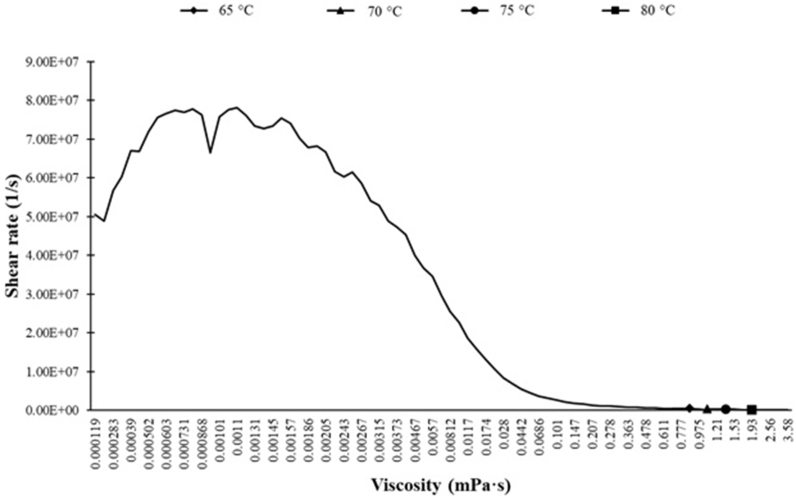
Viscosity of tomato paste versus shear rate and temperature.

The viscosity of tomato paste is measured using the MCR532 rheometer device made by Austria as a function of shear rate and temperature at 65, 70, 75, and 80 °C with the shear rate in the range of 0–90 s^−1^ as depicted in (Fig. 4). These temperatures indicate the operating temperature range of the heat exchanger in the pasteurization process. The Herschel–Bulkley fluid model is used to describe the behavior of the tomato paste due to the existence of yield stress and better tomato paste flow in the heat exchanger in Fluent. The values of k, n, yield stress, and critical shear rate in equation (9), are defined in the material section of the Fluent software.

(9) $$\tau =\tau_{0}+k{\dot{\gamma }}^{n}$$

Where $\tau_{0}$ is the yield stress, k is the consistency factor and n is the flow behaviour index.

#### 1.5.2 Measuring thermal conductivity and specific heat capacity for tomato paste

**Fig. 5:**
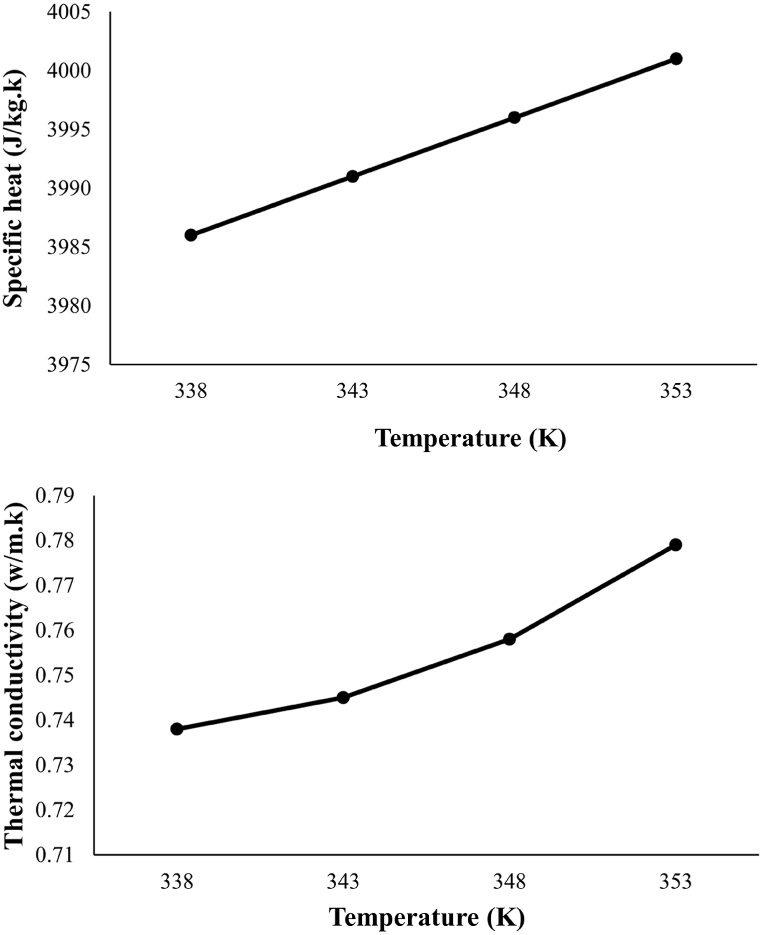
Specificheat and thermal conductivity of tomato paste versus temperature.

As presented in (Fig. 5), the thermal conductivity and specific heat capacity of tomato paste are measured using the thermal properties meter device made by Decagon Company, U.S. These parameters are measured using the sh-1 sensor as a function of temperature at 65, 70, 75, and 80 °C. According to the graph, the thermal conductivity changes and increases with temperature. However, this parameter does not show much variation with temperature for this non-Newtonian fluid. The specific heat capacity of tomato paste is a function of temperature and increases with it, but similar to thermal conductivity, this variation is not significant.

#### 1.5.3 Density

The density of tomato paste is measured using a pycnometer and held constant at a value of 1070 throughout the entire heat exchanger due to the presence of the force by the flow. The specific heat capacity and thermal conductivity are included in Fluent using piecewise-linear functions.

#### 1.5.4 Water properties

The properties of water, which is the fluid inside the shell side, are included in the material section of the Fluent by a piecewise-linear function of temperature using thermodynamic tables [29].

## 2 Discussion and conclusion

### 2.1 Model analysis using thermal results from the tomato paste production line

Computational fluid dynamics is used in this study to simulate heat transfer in the tomato paste pasteurization process. In this stage, the tomato paste enters the tube side of the shell and tube heat exchanger at 65 °C and leaves it at 80 °C. These temperatures are measured using a thermometer in the factory. The results of the CFD simulation of the tomato paste pasteurization process predict a 79 °C temperature at the outlet, which is close to the thermal measurements in the factory. The reason for the difference in the result of simulation compared to the result of temperature data collection can be due to the complex geometry and some assumptions of the solution to achieve optimal conditions for software simulation.

**Fig. 6:**
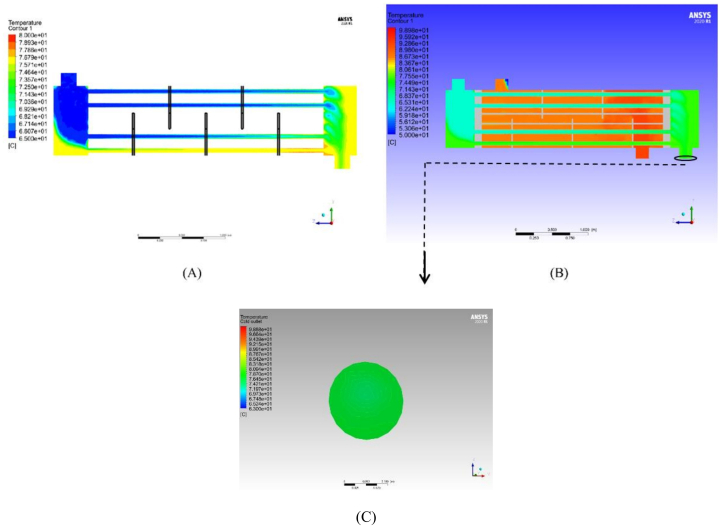
a) Temperature profile of tubes side, b) Temperature profile of tubes side and shell sides, c) Output temperature of tomato paste.

This shows that CFD can simulate other similar processes in various food production lines and is an efficient tool for engineers. In this simulation, an industrial-scale shell and tube heat exchanger with a 3.5 m length, similar to the one in the factory, is imported into the Fluent software. As can be seen in (Fig. 6(a)), the upper tubes of the heat exchanger are cooler than the lower tubes. This temperature difference was also observed in the heat exchanger in the factory. Fig. 6(c) demonstrates heat transfer in the shell side as the hot water flows and loses heat. The effect of neglecting gravity is also evident from this figure because if there exists gravity, the heat is transferred more uniformly at the outlet of the shell side. Fig. 6(c) illustrates tomato paste temperature at the outlet of the heat exchanger. This temperature contour reveals that these regions form around the outlet nozzle. The temperature does not a uniform distribution, and as can be seen in (Fig. 6(c)), there are slightly decentered high-temperature regions at the nozzle cross-section.

**Fig. 7:**
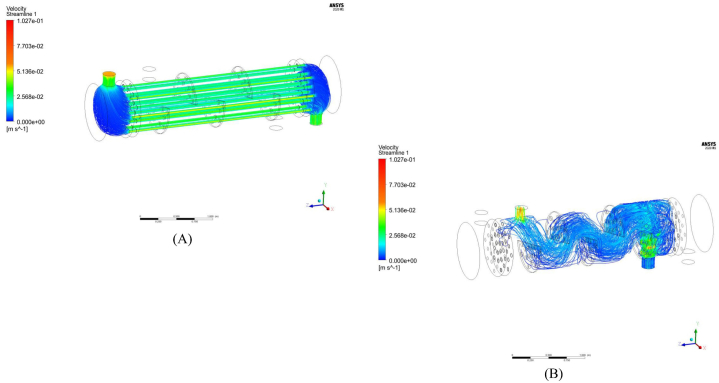
a) Velocity streamline for tubes side, b) Velocity streamline for shell side.

Fig. 7(a) shows the flow and streamlines representing velocity for the tomato paste as a non- Newtonian fluid. As illustrated and according to Ref. [17] the sectional baffles in the shell and tube heat exchanger create a considerable dead zone and swirling flow region. According to the [17] use of helical baffles results in higher thermo-hydraulic performance while trefoil-hole baffles have higher heat transfer performance with large pressure drop compared to segmental baffles.

Fig. 7(a) also shows that the tomato paste, as a fluid that flows much harder than a Newtonian fluid, flows uniformly throughout the entire heat exchanger and tubes to the end. To reach high efficiencies, the fluid flow in the shell side is considered turbulent, which is shown in Fig. 7(b) by velocity streamlines. Fig. 7(b) illustrates that the hot water in its path to the outlet flows completely turbulently. This phenomenon is the result of the Newtonian characteristic of water and the presence of baffles in the flow path. Fig. 7(b) also demonstrates the effect of neglecting the gravity factor in the calculations since the flow through the shell outlet is slanted from the side of the last baffle towards the shell outlet nozzle. According to Ref. [30] this phenomenon reveals the reason behind the high Reynolds number in these regions. The experimental results also confirm these simulation results.

**Fig. 8:**
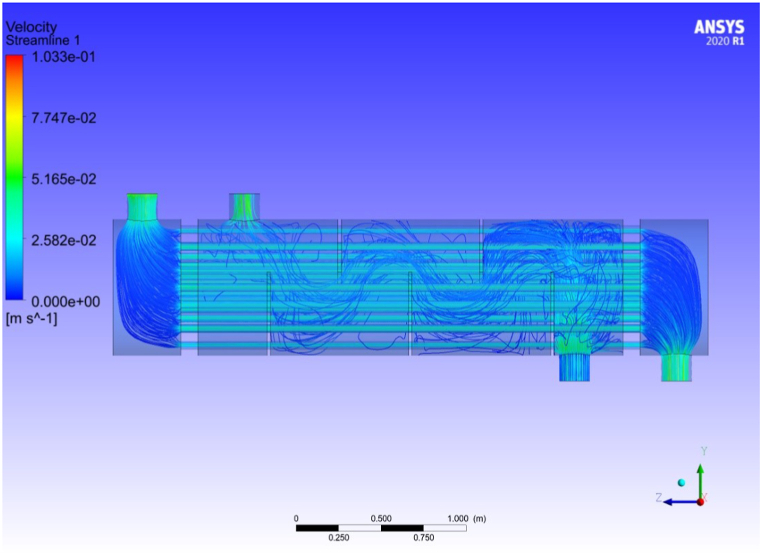
Velocity streamline for tubes and shell.

Fig. 8 illustrates velocity streamlines for both the tomato paste and hot water. Fig. 8 visually demonstrates and confirms the above discussion.

### 2.2 The rheological model describing the tomato paste behavior

Given the Herschel–Bulkley model and yielding stress, which shows that the comparison between this model and the temperature-dependent viscosity model indicates a higher velocity in Newton's law (due to a constant dynamic viscosity and diameter of the tube (dz)) and considering the tube length and fluid density do not change (according to equation number 8), this velocity increase manifests itself in Reynolds number. A comparison between the Reynolds number of this model and the previous one shows a large difference in the Reynolds number values of these models. In CFD, a Reynolds number close to zero indicates flow reversal in tubes. Using the MCR532 rheometer device, values of 11.7 and 0.43 are measured for k and n, respectively, for the tomato paste. Values of 292.17 and 0.241 are obtained for the yield stress and critical shear, respectively. The tomato paste samples show the behavior of a pseudo-plastic non-Newtonian flow with yielding stress [31].

### 2.3 Mesh independency results

Changing the mesh element size does not affect the predicted temperature for the outlet tomato paste. Simulating the heat exchanger with smaller mesh sizes leads to higher computational costs. Table 2 presents the mesh characteristics based on two important measures of aspect ratio and orthogonality for examining the mesh quality.

**Table 2:** Mesh details.

Element size	Nodes	Elements	Mesh structure	Average of aspect ratio	Average of orthogonal
3	1958873	8886046	unstructured	1/8471	0/77183
2.5	1966149	8921720	unstructured	1/8468	0/77179
2	1987538	9023555	unstructured	1/8466	0/77199
3.5	1955585	8868823	unstructured	1/8472	0/77193

### 2.4 Zero or close to zero Reynolds limitation

Given the high viscosity of the tomato paste, the analyses and experimental results reveal that for a better heat exchanger operation, careful attention must be paid to the inlet velocity of the tomato paste because a lower velocity than the measured one may lead to reverse flow in the tubes. This reverse flow can seriously deteriorate the thermal efficiency of the heat exchanger. According to the results of [32], a reverse flow occurs in zero or almost zero Reynolds number flows (Stokes flow), which is in agreement with the results of this study.

### 2.5 Evaluation of the model with different mass flow rates

To reduce costs, avoid spending more energy and reduce pump power, this heat exchanger has been evaluated with three hot water inlet mass flow rates with values of 0.25 kg/s, 0.5 kg/s and 0.75 kg/s. So that the output temperature of tomato paste did not change using three hot water inlet speed rates. This evaluation according to the study of [19] shows that by using three hot water inlet velocity rates used in this study, which are lower than the inlet velocity measured in the factory, the tomato paste reaches the final temperature. So, to avoid spending more energy and because this heat exchanger is simulated with an industrial scale similar to the original model in the factory, the hot water inlet velocity for the heat exchanger should be less than 1 m/s.

## 3 Conclusion

By using a CFD study, a three-dimensional model of a shell-and-tube heat exchanger in an industrial dimension was designed by ANSYS FLUENT commercial software to simulate the heat transfer process in the pasteurization process of tomato paste. The tomato paste enters the tube side of the shell and tube heat exchanger at 65 °C and leaves it at 80 °C The results of the CFD simulation of the tomato paste pasteurization process predict a 79 °C temperature at the outlet, which is close to the thermal measurements in the factory. Due to the presence of yield stress, which was measured and reported, the Herschel-Bulkley model was used to describe the viscosity behavior of tomato paste. It was also shown that the use of a segmental baffle in this heat exchanger is suitable due to the high sensitivity of food to heat compared to other types of baffles such as trefoil-hole and helical baffles, due to lower heat transfer. And that the output temperature of tomato paste did not change using three hot water inlet mass flow rates. So to reduce the pump power, a mass flow rate of 0.25 kg/s was used as the optimal speed for pumping tomato paste into the pipes. In conclusion, the results of this study can be presented for reporting to the industry and factories due to the visualizations of thermal processes such as pasteurization to obtain safe and higher quality food.

## Funding information

This study was supported by the office of vice chancellor for re-search at 10.13039/501100009694Bu-Ali Sina University (Thesis No. 6375).

## Ethical approval

This study does not involve any human or animal testing. Informed Consent: Not require.

## Data availability statement

The data that support the findings of this study are available from the corresponding author upon reasonable request.

## CRediT authorship contribution statement

**S.H. Asadbeigi:** Data curation, Methodology, Software, Writing – original draft. **E. Ahmadi:** Investigation, Project administration, Software, Writing – review & editing. **M. Godarzi:** Methodology. **A. Sagharichian:** Software, Validation.

## Declaration of competing interest

All authors declare that there is no conflict of interest.
